# A Review of Deep Learning Techniques for Lung Cancer Screening and Diagnosis Based on CT Images

**DOI:** 10.3390/diagnostics13162617

**Published:** 2023-08-08

**Authors:** Mohammad A. Thanoon, Mohd Asyraf Zulkifley, Muhammad Ammirrul Atiqi Mohd Zainuri, Siti Raihanah Abdani

**Affiliations:** 1 Department of Electrical, Electronic and Systems Engineering, Faculty of Engineering and Built Environment, University Kebangsaan Malaysia, Bangi 43600, Malaysia; ammirrulatiqi@ukm.edu.my; 2 System and Control Engineering Department, College of Electronics Engineering, Ninevah University, Mosul 41002, Iraq; 3 School of Computing Sciences, College of Computing, Informatics and Media, Universiti Teknologi MARA, Shah Alam 40450, Malaysia; 2022236678@student.uitm.edu.my

**Keywords:** lung cancer, deep learning techniques, detection, diagnosis, classification, segmentation

## Abstract

One of the most common and deadly diseases in the world is lung cancer. Only early identification of lung cancer can increase a patient’s probability of survival. A frequently used modality for the screening and diagnosis of lung cancer is computed tomography (CT) imaging, which provides a detailed scan of the lung. In line with the advancement of computer-assisted systems, deep learning techniques have been extensively explored to help in interpreting the CT images for lung cancer identification. Hence, the goal of this review is to provide a detailed review of the deep learning techniques that were developed for screening and diagnosing lung cancer. This review covers an overview of deep learning (DL) techniques, the suggested DL techniques for lung cancer applications, and the novelties of the reviewed methods. This review focuses on two main methodologies of deep learning in screening and diagnosing lung cancer, which are classification and segmentation methodologies. The advantages and shortcomings of current deep learning models will also be discussed. The resultant analysis demonstrates that there is a significant potential for deep learning methods to provide precise and effective computer-assisted lung cancer screening and diagnosis using CT scans. At the end of this review, a list of potential future works regarding improving the application of deep learning is provided to spearhead the advancement of computer-assisted lung cancer diagnosis systems.

## 1. Introduction

Lung cancer is the leading cause of cancer-related deaths globally [1]. It accounts for 18% of all cancer-related deaths, making it the most common cause of death among all cancers. The major cause of lung cancer is smoking, and it has reached a peak or is continuing to rise in several countries. This suggests that lung cancer will become more common for at least a few decades [2]. Early detection and accurate diagnosis of lung cancer can significantly improve patient outcomes [3,4]. Individuals with lung cancer have a 10- to 20% probability of surviving five years following diagnosis. Computed tomography (CT) and magnetic resonance imaging (MRI) are common medical procedures for early detection that improve patient survival [5,6]. The best features can be produced manually using feature extraction methods like Sequential Flood Feature Selection Algorithms (SFFSA) or Genetic Algorithms (GA), which have historically been the foundation of intelligent procedures [7].

**Figure 1:**
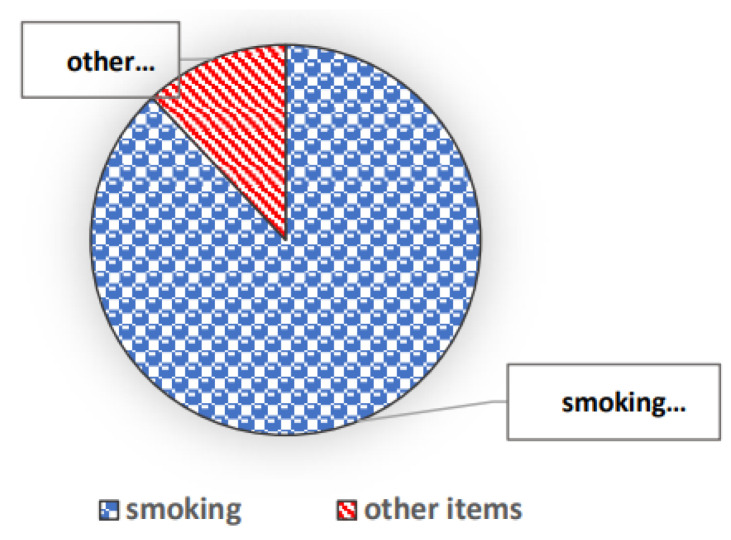
Causes of lung cancer [9].

Recent advances in deep learning technology have enabled CAD systems to autonomously identify visual features [8]; as a result, many medical image-processing techniques have been successfully applied [9]. The two main subtypes of lung cancer are small-cell lung cancer (SCLC) and non-small-cell lung cancer (NSCLC). Generally, lung cancer is caused by several factors, including smoking, harmful airborne particles, sex, genes, aging, and others [10]. Long-term smoking is the main contributor to lung cancer, as shown in Figure 1 [11]. Smoking causes lung cancer not just in smokers but also in secondhand smokers. Some of the signs that can be used to screen for lung cancer are yellow fingers, anxiety, chronic illness, tiredness, reactions to allergens, wheezing, roaring, coughing up blood (even for minor amounts), hoarseness, breathing problems, pain in the bones, headaches, swallowing problems, and chest discomfort [12].

In general, early detection of a cancer case through accurate diagnosis followed by effective treatment can increase the likelihood of a full cure [13]. Regardless of the medical tools, authorized specialists are required for the interpretation of medical information for the diagnosis of illnesses. It is also a norm that experts sometimes have disagreements due to the complexity of medical images. Therefore, an intelligent, assisted diagnosis system is essential in the medical field. In recent years, conventional machine learning (ML) and deep learning (DL) algorithms have been used to analyze and interpret medical images for diagnosis purposes [14]. The development of a computer-assisted diagnosis system for medical applications is extremely challenging, and much research and funding have been granted for various diseases.

The goal of this paper is to provide a thorough overview of the state-of-the-art literature on deep learning algorithms used for classifying and segmenting CT images for lung cancer. This study examines various deep learning methods that have been employed for lung cancer detection and diagnosis and provides a general performance comparison between the benchmarked methods. This paper also highlights the issue of the lack of reliable and effective strategies for the reviewed CT-based intelligent diagnosis system. The common weaknesses of current systems are that they are time-consuming, expensive, and require specialized knowledge. By using a deep learning approach, the intelligent system can provide automatic and precise lung cancer detection and diagnosis using CT images, which is an exciting approach to this issue. This paper also summarizes the advantages and disadvantages of various deep learning approaches and offers suggestions for further research in this area. The ultimate objective is to use deep learning methods based on CT images to increase the precision and efficacy of cancer detection and diagnosis.

### 1.1. Deep Learning Methods Overview

Correctly acquiring medical images and interpreting them is crucial to correctly identifying and diagnosing malignant diseases. There are numerous high-resolution image capture devices available, including CT, MRI, and X-ray scans. After pre-processing, the illness identification system extracts pertinent information from these medical images and uses those features to train its models. A further application of the trained model is to identify the disease from corresponding unknown medical images.

The traditional machine learning method is unable to produce reliable findings since medical images of various individuals vary greatly. Deep learning techniques have been successfully applied in a variety of disciplines recently, particularly in the analysis of medical pictures. These techniques are useful and effective for evaluating medical images to find disorders, especially cancer.

Techniques for deep learning are a subset of machine learning techniques that enable estimating the outcome using the provided data set and training the model based on the outcome. Deep learning techniques use neural networks with numerous layers, including an input layer, several hidden layers, and an output layer. The deep learning model is taught more precisely because there are numerous layers present. Based on their learning methodologies, deep learning models can be divided into four groups: reinforced learning models, unsupervised learning models, semi-supervised learning models, and supervised learning models.

#### 1.1.1. Supervised Deep Learning Models

For the training purposes of this type of deep learning model, specified, labeled data is required. The deep learning approach must be practiced throughout the training phase using every conceivable input combination that includes the target class label. Following training, the model that was developed is used to forecast the label of unidentified samples. CNN, LSTM, RNN, and GRU are the most widely used deep learning models in the supervised learning category.

#### 1.1.2. Unsupervised Deep Learning Models

Deep learning models that have been improved do not need any tagged practice data. To group similar data, these models analyze the data’s inherent characteristics based on some pertinent characteristics. These models are typically employed for feature reduction and clustering reasons. Auto-Encoders (AE) and Restricted Boltzmann Machines (RBM) are two of the most commonly utilized unsupervised deep learning models.

#### 1.1.3. Semi-Supervised Deep Learning Models

For training, a semi-supervised deep learning model uses both labeled and unlabeled data. RNN, LSTM, GRU, and Generative Adversarial Networks (GAN) are the deep learning models in this category that are most frequently utilized.

#### 1.1.4. Reinforced Deep Learning Models

Reinforced deep learning models focus on selecting the best course of action to maximize rewards in a certain environment. These models learn to recognize real behavior by interacting with the outside world.

**Figure 2:**
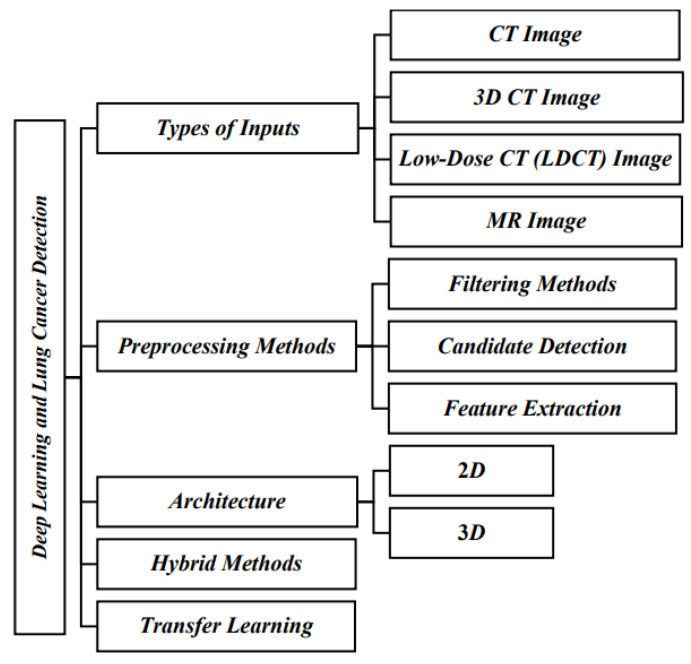
Overall stature of lung cancer detection.

Deep learning is a branch of the machine learning field that makes use of multiple-layered, complex networks to learn features of interest from vast amounts of data. These networks can extract the linkages and patterns within the data that are too complicated for conventional machine-learning techniques to comprehend. Due to its capacity to automatically learn complex characteristics from a variety of input medical image sources, such as 2D CT pictures, 3D CT images, low-dose CT images, and MR images, as illustrated in Figure 2, deep learning techniques have grown in popularity as the methods used in lung cancer diagnosis.

As CT scans offer highly detailed images of the lungs, they are frequently employed in the identification of lung cancer. By examining the size, shapes, textures, and intensities of the pictures, deep learning algorithms are able to recognize the affected nodules based on the CT images. Since they provide complete imaging of the lung capacity, 3D CT pictures offer a more comprehensive examination of the lungs compared to 2D CT images. In many previous works, deep learning methods have been used to analyze 3D CT images accurately to detect nodules and other abnormalities. To minimize the exposure to radiation during lung cancer screening, low-dose CT methods are the preferred modality, coupled with denoising and image augmentation techniques. Compared to CT scans, MR imaging has more capability for revealing information about tissue density and blood flow. Based on their distinctive features, deep learning systems can also analyze MR images to locate lung nodules along with additional abnormalities. To increase image quality and reduce noise, filtering techniques such as Gaussian and Median filtering are usually implemented to pre-process the incoming images. In lung cancer cases, candidate detection is the process of spotting areas of concern in the image to detect possible nodules or additional abnormalities. There are several methods in deep learning that can be used to identify prospective candidates, including region proposal networks and sliding window approaches.

In general, 2D deep learning algorithms such as recurrent neural networks (RNNs), CNNs, and hybrid models have been frequently employed for lung cancer diagnosis due to their ability to examine individual CT slices to detect nodules and other anomalies. Apart from 2D techniques, 3D deep learning architectures are also used to analyze the whole CT volumes to locate the nodules, especially on 3D CNNs, as well as additional models that are normally used for processing 3D data. In order to increase the precision of lung cancer detection, some methods applied a hybrid approach that incorporated various types of deep learning architectures for both 2D and 3D CNN to analyze both individual CT data slices and full volumes. To speed up the training process, many deep learning algorithms examine fresh data using transfer learning methods that pretrain the models using massive datasets of CT images before fine-tuning the models for any specific disease.

### 1.2. Analysis of a CT Image Using DL

CT imaging produces detailed images of the human body, and it has been used as a non-invasive method for diagnosing and monitoring a number of diseases, including cancer, heart disease, and trauma. CT imaging has revolutionized the contemporary medicine field by providing specialists with accurate tools to provide internal organ images of the body, enabling them to make more informed diagnoses and treatment decisions. The attenuation of X-rays as they pass through various body tissues is the basic principle by which CT imaging maps the internal organs. A CT scan can be captured by requesting that the patient be positioned on a table that slides into a large, doughnut-shaped CT scanner. The scanner then emits a number of X-rays that go through the body before being detected by a number of detectors on the scanner’s opposite side. The X-ray attenuation data is then processed by a computer to provide cross-sectional images of the body that can then be further transformed into 3D images. There are a number of advantages of CT imaging compared to other medical imaging modalities, whereby the former imaging technique is non-invasive since it does not involve any invasive treatments or incisions. A standard CT scan also requires a short amount of processing time, making it a rather speedy technique. Furthermore, a CT scan provides the imaging data inside the body in great detail, providing medical professionals with access to irregularities that might not be visible if traditional imaging techniques are used. CT imaging has, however, a number of disadvantages, including the use of ionizing radiation that could eventually increase the risk of getting cancer. Therefore, it should only be used when it is absolutely essential, and it must be administered with the appropriate radiation protection. Furthermore, CT imaging may detect abnormalities that are not actually there, a condition known as false-positive results. This could lead to unnecessary, expensive, and potentially harmful testing and treatment.

CT imaging-based lung cancer diagnosis has improved using DL methods. Deep learning uses multiple-layered networks to learn from enormous volumes of data. These networks are capable of identifying connections and patterns in data that are too complex for traditional machine-learning algorithms to grasp. Deep learning systems can be finetuned to take CT images as input to identify lesions, tumors, and nodules automatically. Additionally, they can be used to segment the images into several anatomical components, such as the liver, brain, and lungs. Medical professionals may be better able to diagnose anomalies in particular body parts thanks to this. The use of deep learning for CT image processing has a number of benefits. Deep learning algorithms can adapt to varied input data types, such as CT images with varying resolutions, noise levels, and contrast, and can learn from vast amounts of data. They can also perform real-time image analysis, which can boost the effectiveness of the diagnostic procedure. The accuracy and consistency of CT imaging analysis can also be improved by deep learning algorithms, lowering the possibility of false-positive and false-negative outcomes.

Deep learning for CT image analysis has a number of difficulties as well. To train deep learning algorithms, big and high-quality datasets are required, but effective data collection is a big hurdle for medical image applications. These datasets must be accurately labeled and annotated to ensure that the algorithms discover the relevant characteristics and patterns. In general, deep learning algorithms are complex in nature and hard to physically interpret. Despite these difficulties, deep learning has the potential to enhance the precision and effectiveness of CT image-based diagnosis. It has the potential to revolutionize the area of medical imaging by giving doctors more precise and dependable diagnostic equipment. To completely assess the efficiency and security of deep learning techniques for CT image interpretation and to address the difficulties involved in their application in clinical settings, a comprehensive review is required.

### 1.3. Paper Organization

This review paper is organized into eight sections, beginning with an introduction that gives a general overview of the application of deep learning algorithms for lung cancer diagnosis using CT images. Section 2 briefly describes deep learning techniques and their application to CT scans. While Section 3 offers a comparative analysis of various deep-learning models used for lung cancer detection, Section 5 and Section 6 discuss the segmentation and classification algorithms used for lung cancer detection, respectively. Section 8 offers concluding observations and recommendations for future study.

### 1.4. Contributions to the Survey

The important contributions of this review paper are as follows:

- Giving a thorough analysis of how deep learning methods are used to identify and diagnose lung cancer from CT scans.
- Summarizing the most popular deep learning models for detecting and classifying lung cancer.
- Comparison analysis of the effectiveness of various deep learning models.
- Outlining the shortcomings of the current approaches and recommending potential study areas.

## 2. Lung Cancer Detection Using Deep Learning Techniques

**Figure 3:**
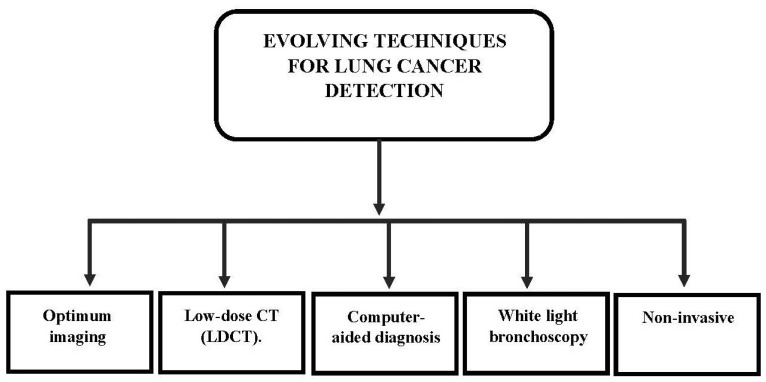
Techniques for lung cancer detection.

**Figure 4:**
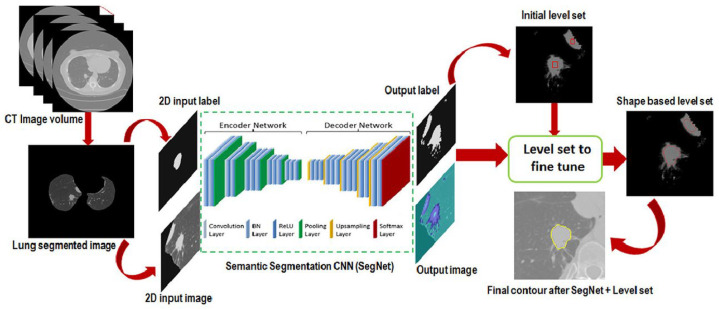
Deep learning algorithm for lung nodule segmentation [15].

The diagnosis process of some diseases can be assisted by using automation methods through Computer-Assisted Diagnosis (CAD). This method utilizes software to segment, predict, locate, and classify the symptoms, which will be used to infer the presence and severity of the diseases. In this work, the focus is to provide a review of CAD methods that are used to identify cancerous nodules in lung CT scans. Generally, a CT scan allows doctors to identify the presence of lung cancer nodules, especially when the nodules are large, which corresponds to the late stage of the disease. However, it is important to identify the nodules at an early stage, which is usually small in size before a patient develops a lung tumor the size of a golf ball. Based on the two images in Figure 3 and Figure 4, it is really difficult to distinguish and segment the nodules manually.

A particular kind of neural network that is excellent for image categorization is the convolutional neural network (CNN). The functioning of the human visual brain served as inspiration for this architecture. A neuron mechanism will be mimicked by a CNN filter with a set of receptive fields that only process a limited portion of the image. Each deeper layer of these neurons has a bigger receptive field and, as such, can learn and recognize more complex patterns, which are primarily arranged in hierarchical layers. CNNs can also be seen as numerous layers of sliding windows with tiny neural networks striding across the image.

One of the advantages of CNNs is their location invariance capability, which allows the filters to learn the patterns regardless of where they are located. This is because the use of sliding windows allows the filter to learn the patterns all over the images. Another advantage of CNNs is their hierarchical structure, which enables them to automatically pick up on increasingly abstract patterns. The first layers may pick up on boundaries and structures, while the intermediate layers may pick up on details like shapes, and even higher levels may pick up on general object shapes.

CNNs can be configured to process 3D images rather than the sliced 2D images of the CT scan. 3D CNNs can be implemented by using a sliding cube instead of a sliding pane that advances through three dimensions while extracting features at each step.

## 3. Computer-Assisted Lung Cancer Detection Using CT Images

Several studies have investigated the use of deep learning algorithms for CT-based lung cancer screening and diagnosis. In general, there are unique image attenuation patterns in CT images for healthy and unhealthy scans. In order to distinguish the lungs from the surrounding tissues, straightforward techniques such as numerical approaches, gray-level thresholding, and shape-based approaches can be used to perform simple lung segmentation.

In [16], Brown et al. proposed an automated, knowledge-based method for segmenting the chest region. Several pieces of anatomical knowledge, including the estimated volume, shape, relative position, and X-ray attenuation of organs, are required inputs for this technique. In [17], Brown et al. further developed a knowledge-based, automated segmentation system to extract useful data from the CT image data. They were able to automatically generate indirect quantitative measurements of single lung function that are inaccessible through traditional pulmonary function tests. Furthermore, a fully automatic method for lung segmentation from 3-D pulmonary X-ray images was developed by Hu et al. [18]. The suggested algorithm’s performance was evaluated using 3-D CT data sets from eight healthy people, and it was discovered that there is a 0.8-pixel difference in the root mean square performance between computer and human analysis. Using a slice-based pixel-value threshold as well as two sets of classification criteria based on size, circularity, and location-related data, the work in [19] proposed a fully automated lung segmentation approach. Based on the test on 101 CT cases, they managed to produce 94.0% segmentation accuracy using 2969 thick slice images and 97.6% segmentation accuracy using 1161 thin slice images. Moreover, the work in [20] introduced an anisotropic filtering methodology and a wavelet transform-based interpolation method for segmenting and visualizing lung volume. Using single-detector CT scans, the suggested method’s robustness and efficacy were shown through the percentage improvements in volume overlap and volume difference. 

In [21], Swierczynski et al. described a segmentation method that merged a conventional segmentation method with active dense displacement field estimation based on a level-set formulation. The findings showed that the suggested approach was more accurate than the independent registration and segmentation processes. Based on a universal lung nodule shape model, the work in [22] proposed a new variational level set approach for lung nodule segmentation using CT scan data. The suggested method has the benefit of being independent of the type or location of nodules. Using a similar idea, the work in [23] proposed a parameter-free segmentation technique with an emphasis on juxtapleural nodules to increase lung nodule identification accuracy. The 403 juxtapleural nodules from the lung imaging database consortium (LIDC) were used to demonstrate a re-inclusion rate of 92.6%. Apart from that, Zhang et al. [24] created a worldwide optimal hybrid geometric active contour model and an automated lung segmentation method. The incorporation of global region and edge information increases the algorithm’s accuracy in areas with weak boundaries or narrow bands. The segmentation method described by [25] starts with a sphere inside the segmentation target lung and deforms it further as forces are applied toward the lung borders. The algorithm’s effectiveness was demonstrated using 40 CT scans, and the average F-measure is 99.22%.

Researchers have been investigating the convolutional neural network’s robustness for computer vision applications for the past ten years. For natural image processing and medical image analysis, various CNN-based methods have been proposed. The use of artificial intelligence and CT scans to identify and diagnose lung cancer has been suggested in a number of ways. To recognize and categorize lung nodules, for instance, the work in [26] created a three-dimensional convolutional neural network (CNN) with three modules. This technique beats manual assessment performance with a sensitivity rate of 84.4%. Similarly, Nasser and Naser [27] detected lung cancer using an artificial neural network (ANN) with a high accuracy of 96.67%. Cifci et al. [28], the suggested Deep Learning with Instantaneously Trained Neural Networks (DITNN) method, coupled with the enhanced profuse clustering technique (IPCT), managed to improve lung image quality and the diagnosis rate of lung cancer with 98.42% accuracy. With an accuracy performance of 0.909 and 0.872, respectively, the paper in [29] suggested a double convolutional deep neural network (CDNN) and a conventional CDNN to recognize and classify lung nodules. 

Uniquely, Wang et al. [30] created a computer-aided detection (CAD) system that had low false negative and positive detection rates while achieving excellent nodule detection accuracy. Rather than using random initialization, the deep model in [31] classified lung pictures using an inception-v3 transfer learning method and managed to attain a sensitivity rate of 95.41%. Last but not least, the approach in [10] suggested a multi-group patch-based learning system that could detect lung cancer with a sensitivity rate of 80.06% with 4.7 false positives per scan and a sensitivity rate of 94% with 15.1 false positives per scan. In [32], the authors designed a dense convolutional binary-tree network (DenseBTNet) with high parameter efficiency as well as multi-scale feature extraction capabilities. In their work [33], Li et al. highlighted the significance of early detection in lowering lung cancer mortality. They unveiled a deep learning-based computer-aided diagnosis (DL-CAD) system that can identify and categorize lung nodules with a diameter of less than 3 mm while also estimating the likelihood of these nodules developing into malignancy types. The system’s sensitivity was tested using the LIDC-IDRI and NLST datasets, and it achieved 86.2% accuracy.

Similar to the previous approach, the work in [34] presented a deep 3D residual convolution neural network with a focus on lowering false positives for the automated detection of lung nodules in CT scans. They utilized a spatial pooling and cropping (SPC) layer to extract multi-level contextual data, and their 27-layer network utilized the LUNA-16 dataset to obtain a sensitivity rate of 98.3%. Teramoto et al. [35] described a new Deep Convolutional Neural Network (DCNN) including convolutional, fully connected, and pooling layers for the automated classification of lung cancer. A small dataset of 76 cancer cases was used to train the DCNN, which had only 71% classification accuracy. A 3D convolutional neural network was also suggested by [36] for computer-aided pulmonary nodule diagnosis from volumetric CT scans. Their model, which was tested using the LUNA16 dataset, consists of several sets of 3D convolutional layers, max-pooling layers, fully connected layers, and a softmax layer. Their findings show that 3D CNNs can improve detection accuracy; they achieved a sensitivity rate of 94.4%.

Based on CT images, two works (i.e., [37,38]) applied deep learning algorithms to predict lung adenocarcinoma survival rate as well as EGFR mutation status and its subtype categorization. To recap the outputs of several works, a review was conducted on the application of deep learning methods for CT imaging lung nodule segmentation and classification [39]. Contrary to the high-dose technique, the work in [40] used low-dose chest CT images and a three-dimensional deep-learning model to create an end-to-end lung cancer detection system. Furthermore, Shao et al. [41] have applied deep learning algorithms to mobile low-dose CT scans as a screening tool for lung cancer in resource-constrained locations. Similarly, based on CT scans, the model in [42] is used to forecast the EGFR mutation as well as the PD-L1 expression status in non-small-cell lung cancer, and a thorough analysis of the use of these deep learning techniques for CT scans for lung nodule detection and diagnosis can be found in [43].

Apart from the classification approach, deep neural networks were also employed by [11] to segment lung CT images. Lakshmanaprabu et al. [44], the suggested deep learning model produced the best performance for classifying lung cancer from CT data, with a 96.3% accuracy rate. As an exploration work, Lee et al. [45] examined how deep learning models are used in chest radiography and how CT images are utilized to identify lung cancer and emphasized how these models have the potential to increase precision and effectiveness in clinical settings. As an example, the work in [46] described a deep learning method for lung cancer diagnosis that had a 93.55% sensitivity and 91.5% specificity. For a smaller nodule case, the model developed in [47] assessed PD-L1 expression in non-small cell lung cancer and predicted immune checkpoint inhibitor responses using deep learning on CT images. With an F1 score of 0.848, Hu et al. [48] suggested a deep learning method for automatically extracting information on the stage of lung cancer from CT data. To further prove the effectiveness of a deep learning-enhanced computer-aided diagnosis (CAD) system in identifying lung nodules on 1-mm-thick CT images, the works in [49,50] suggested a machine learning method that could identify benign, preinvasive, and invasive lung nodules on CT scans. A deep learning system for lung cancer prediction was also put forth by [51], with an accuracy rate of 87.63%.

Vani et al. [52], the authors propose six deep learning models (CNN, CNN GD, Inception V3, Resnet-50, VGG-16, and VGG-19) for efficient lung cancer diagnosis using CT scans and histopathological images. Evaluation based on accuracy, F-Score, precision, sensitivity, and specificity shows CNN GD outperforms others with 97.86% accuracy, 96.39% accuracy, 96.79% sensitivity, and 97.40% specificity. Shalini et al. [53], the authors propose a novel approach using 3DCNN and RNN for classifying cancerous lung nodules with high accuracy (95%). Future enhancements could involve big-data analytics and cascading classifiers to further improve efficiency. Abunajm et al. [54], the authors introduce a CNN-based model for early prediction and detection of lung cancer from CT scan imaging, capable of identifying benign, malignant, and normal cases. Detecting lung cancer early is vital for improving survival rates and initiating timely treatment plans. The model successfully reduces false positives and achieves an impressive accuracy rate of 99.45%.

A combination of radiomics and deep learning methods was also proposed in [55] to detect and manage lung cancer. In order to enhance cancer detection and prognosis, the scientists describe how radiomics can extract quantitative data from medical images. This data may then be analyzed by deep learning algorithms. A study on the application of deep learning to forecast the risk of cardiovascular disease from low-dose computed tomography (CT) images used for lung cancer screening is presented in [56]. To forecast the risk of cardiovascular illness based on lung CT scans, the researchers developed a deep learning algorithm on a huge dataset of various cardiovascular risks. The algorithm developed in [57] suggested a more effective method for detecting lung cancer from CT scans using hybrid dense clustering and deep learning to instantly train the neural networks. The authors demonstrated the efficacy of their technique in identifying lung nodules and compared it to other methods of identifying lung cancer. A unique deep convolutional neural network (CNN) is presented in [58] for the diagnosis of lung nodules on 3D CT images, which was tested on a sizable dataset of CT scans to show the detection and categorization of the lung nodules. Zhao et al. [59], the authors put forth a weighted discriminative extreme learning machine concept for electronic nose system lung cancer diagnosis. They obtained great accuracy in differentiating between the two groups by analyzing breath samples from lung cancer patients as well as healthy controls using an electronic nasal device. Using 18 F-FDG PET/CT images, Chen et al. [60] offers a multimodality attention-guided 3D detection approach for non-small cell lung cancer. The accuracy of lung cancer detection in PET/CT scans was improved by the authors using deep learning algorithms, which can help with the early diagnosis and treatment formulation of lung cancer. Table 1 summarizes the applications, pros, and cons of different lung imaging methods, while Table 2 summarizes the reviewed models of the past 5 years (2018–2022).

## 4. Dataset Discussion

The literature survey on lung cancer detection has utilized various datasets to evaluate the performance of deep learning techniques. Some of the datasets that have been employed in the research include:

Level-set approach for joint image segmentation and registration and CT image dataset: This dataset is used in studies exploring joint image segmentation and registration techniques to enhance lung cancer detection accuracy.

- Lung Image Database (LID): The Lung Image Database is often used to develop and validate computer-aided diagnosis systems for lung cancer based on deep learning algorithms [62].
- LIDC-IDRI: The Lung Image Database Consortium and Image Database Resource Initiative dataset is a widely used dataset for lung cancer research, providing annotated CT images for nodule detection and classification tasks [63].
- NLST: The National Lung Screening Trial dataset is commonly used for low-dose CT imaging research to evaluate the performance of deep learning models in early lung cancer detection [64].
- ImageNet: ImageNet is a vast dataset used for pretraining deep learning models. It has been utilized in transfer learning approaches for lung cancer detection tasks [65].
- Immunotherapy dataset: This dataset has been employed to investigate the relationship between lung cancer and immunotherapy responses using deep learning methods [66].
- PD-L1 expression dataset: The PD-L1 expression dataset has been utilized to explore the use of deep learning in predicting PD-L1 expression levels in lung cancer patients [47].
- Tianchi AI dataset: The Tianchi AI dataset has been used in various studies to develop and evaluate deep learning-based lung cancer detection systems [67].
- CT lung datasets: Different CT lung datasets are used in studies focusing on lung-specific image analysis and nodule detection using deep learning methods [68].
- Cancer Imaging Archive (CIA) dataset: This dataset is commonly used in research to develop and assess deep-learning models for lung cancer diagnosis [69].
- Private datasets: Some studies have utilized private datasets, the sources of which are not publicly disclosed, to evaluate the performance of deep learning techniques [70].

These datasets vary in size, imaging modalities, annotation quality, and diversity of lung cancer cases, which poses both opportunities and challenges for developing accurate and generalizable deep learning models.

Table 2 summarizes the reference papers used in the literature survey (latest 5 years, 2018–2022) (Section 3).

## 5. Evolving Techniques for Lung Cancer Detection

One of the cancers that leads to mortality is lung cancer. Furthermore, it is very challenging to identify the cases because they normally emerge and manifest during the terminal stage. However, early disease detection and treatment tools can lower the mortality rate. Optimum imaging methods such as CT imaging may reveal all suspected and undetected lung cancer nodules, which makes it a trustworthy tool for diagnosing lung cancer [71]. It may be challenging to identify the malignant cells, nevertheless, due to variations in CT scan intensity and anatomical structure misinterpretations by medical professionals and radiologists [72]. Computer-aided diagnosis systems have recently become popular tools to help radiologists and clinicians effectively diagnose cancer [73]. Numerous systems have been created, and research into the detection of lung cancer is still ongoing. However, some systems still need to be improved in order to obtain the best detection accuracy possible, which is going towards 100%.

Lung cancer can be effectively treated with the help of a thorough etiology, reliable early identification, and appropriate medications. Thus, early detection of lung cancer is essential, particularly in screening high-risk populations (such as smokers, those exposed to fumes, those working in oil fields or other toxic environments, etc.) with a pressing need to find new biomarkers. Furthermore, the best lung cancer treatment relies on the precision of the diagnostic tools. Therefore, the need to find sensitive and precise biomarkers for early detection is crucial, and current methods perform lung cancer screening procedures by using low-dose CT (LDCT). Additionally, compared to no screening cases, a study (NELSON) [74] has demonstrated that this specific screening method has a selectivity of 85% and a specificity of 99%. Moreover, according to a recent study [75], the overall false-positive rate was actually less than 81%, which needs to be confirmed by further imaging or testing due to this extremely high number.

A quick description of lung cancer staging is provided below to clarify the timing of the screening with respect to the development of lung cancer. Small-cell lung cancer (SCLC) and non-small-cell lung cancer (NSCLC) are the two main subtypes of lung cancer. SCLC is a central tumor that manifests as a perihilar mass in the airway submucosa. According to histological investigations, the neuroendocrine cells of the basal bronchial epithelium are the source of this particular type of cancer. Small, spindle-shaped, or rounded cells with little cytoplasm, granular chromatin, and necrosis are the most typical cell types found in this case [76]. The subtypes of SCLC include pure and mixed NSCLC, which can be distinguished from the possibility of brain, liver, and bone metastases [77] and is categorized as having limited or widespread stages [78]. Either the ipsilateral mediastinum, mediastinal, or supraclavicular lymph nodes and one radiation site detected can be used to identify the restricted SCLC stage. As long as it is present on the same side of the cancerous chest, it is regarded as belonging to the category of supraclavicular lymph nodes. On the other hand, widespread SCLC spreads to the second lung lobe, lymph nodes, and additional organs like the bone marrow and is not constrained to a single radiation point in the lung.

The likelihood of brain, liver, and bone metastases can be used to distinguish this type of cancer [79], and it is defined as having restricted or widespread stages [80]. The confined SCLC stage includes the ipsilateral mediastinum, mediastinal, or supraclavicular lymph nodes at one radiation site. It is considered to be part of the supraclavicular lymph nodes as long as it is present on the same side of the cancerous chest. On the other hand, widespread SCLC is not limited to a single radiation point in the lung; it can spread to the second lung lobe, lymph nodes, and extra organs such as the bone marrow. In terms of sensitivity, chest radiography is less sensitive compared to a chest CT scan, but it produces more detailed imaging. Given these traits, creating a computer-aided diagnosis (CAD) model for chest radiographs would be beneficial since it would increase the detection sensitivity while preserving the low false-positive (FP) findings [81].

The cytological study of sputum, particularly several samples, is another method of diagnosing lung cancer, and it usually aids in the discovery of a core tumor in the bigger bronchi. In general, small adenocarcinomas with a diameter less than 2 cm that originated from airway ramifications, such as small bronchi, bronchioles, and alveoli, were not seen in sputum samples [82]. This information has gained more significance recently as adenocarcinomas and squamous cell carcinomas have increased and decreased, respectively, in response to changes in cigarette exposure. According to a few screening studies, the sensitivity of sputum cytology for early lung cancer is only in the range of 20–30%. Early research indicated that several parameters, including the number and kind of cells (deeper airways), can affect the ability to detect pre-malignant situations [83]. Sputum cytology has also been found to be insufficiently sensitive or accurate for routine workup of any patient that is suspected of having lung cancer, according to [84]. The most popular diagnostic method for analyzing a certain histological lung cancer diagnosis is white light bronchoscopy (WLB). For pre-malignant lesions, bronchoscopy has substantial diagnostic capability. In general hospitals, a method based on tissue biopsy is the gold standard for confirming the presence of malignancy. To use histopathological techniques to determine the subtype of lung cancer, lung tissue biopsy samples must be large enough. Early diagnosis must be confirmed by the initial biopsy in order to prevent repeated procedures, which run the risk of complications and delay the start of treatment. In order to diagnose lung cancer, a variety of techniques are frequently used, such as fiber optic bronchoscopy with or without transbronchial needle aspiration, endobronchial ultrasound, image-guided trans-thoracic needle aspiration, mediastinoscopy, pleural fluid analysis (thoracentesis), thoracoscopy, and surgical methods. These techniques are known to be costly and prone to errors, and many samples may be required [85].

The capacity to diagnose peripherally tiny tumors has been improved with the introduction of spiral CT images. These images, however, have a much poorer sensitivity for tumor detection that is more centrally situated (mainly squamous cell carcinoma) than for tumors that are more peripherally located [86]. Notably, 96% of all positive screens in the LDCT of the National Lung Screening Trial were found to be false positives, as measured by over 40% of all individuals [64] with at least one positive screen. Furthermore, for smokers who are cancer-free, the high-frequency screening of false-positive results in expensive and invasive procedures. In order to diagnose lung cancer, it is highly important to screen for the disease with less expensive equipment and non-invasive methods.

Recent advances in radiology have been made possible by the use of convolutional neural networks (CNN), a branch of deep learning (DL) techniques [87,88]. The detection of nodules and masses on chest radiographs has also shown promising results when DL-based models are used, which have reported sensitivities in the range of 0.51–0.84 and mean numbers of FP indications per image (mFPI) of 0.02–0.34. Additionally, these CAD models managed to improve radiologists’ ability to detect nodules compared to screening procedures without them. It might be difficult for radiologists to locate the nodules and distinguish between benign and malignant nodules [89,90]. Radiologists must also pay close attention to the shape and marginal characteristics of nodules since normal anatomical structures do resemble healthy nodules. Even competent radiologists can make a mistake in performing the diagnosis because these issues are condition-based rather than radiologist-related [91,92]. Segmentation and detection are the two primary DL approaches for lesion identification. In contrast to the segmentation method, which classifies pixels, the detection method classifies a region as a single label. Compared to the detection approach, the segmentation method can offer more precise information that goes down to each individual pixel’s label. In clinical practice, pixel-level classification of a lesion’s size can improve the likelihood of a successful diagnosis. Since the shape can be used as a guide during the detection process, pixel-level categorization will make it simpler to monitor changes in the lesion size and shape. It also provides information on the area of the lesion in addition to the long and short diameters when evaluating the treatment’s effectiveness [93]. Figure 3 shows the techniques for lung cancer detection.

### Research Gaps and Challenges

- Early diagnosis of lung cancer is crucial to improving survival rates, but it remains challenging due to factors like low contrast variation, heterogeneity, and the visual resemblance between benign and malignant nodules in CT images [94].
- Accurately detecting lung nodules in medical imaging is difficult due to the intricate lung anatomy and the need for labeled samples, which can be time-consuming to acquire [95].
- Deep learning algorithms have shown promise in automatically identifying features in lung nodule CT images, but their performance is often compared to traditional computer-aided diagnosis (CADx) systems that rely on hand-crafted features [96].
- There is limited research on utilizing Convolutional Neural Networks (CNNs) to analyze EBUS images, and distinguishing between benign and potentially cancerous tumors based solely on EBUS images is challenging [97].
- Some studies have focused on predicting mortality risks based on CT scans of NSCLC patients but failed to identify early-stage lung or lobe-related malignant lesions [98].
- Understanding how CNNs predict the malignancy of a specific nodule and the importance of the region within a nodule or contextual information in the CNN’s output remains unclear [99].
- Computer-assisted lung disease diagnosis is essential due to noise signals that degrade the quality of cancer images during the picture capture process [100].
- The diverse appearance of different lung nodules and the scarcity of positive samples in available datasets pose challenges for training Deep Convolutional Neural Networks (DCNNs) [101].

## 6. Segmentation Process

Image segmentation is a technique used to identify the outlines of an organ’s or anatomical structure’s outlines. The applied deep learning techniques have shown significant improvement in the semantic segmentation task, which makes them a powerful tool for medical diagnosis. This technique entails identifying the organs or lesions in medical imaging, such as CT or MRI, which provide important details on the sizes and shapes of these organs [102,103]. In the literature, a number of automatic segmentation systems have been proposed by various researchers. However, conventional methods like edge detection and mathematical-based filters were frequently used as the pre-processing step. Furthermore, methods using deep machine learning to extract complex features have been the way to go. The design and extraction of the hand-crafted features were the key concerns for creating such a system, and the deployment was severely constrained by the complexity of these procedures. Deep learning capabilities for image processing, especially for image segmentation, have been heavily used by medical researchers and include various modalities of 2D CNN, 2.5D CNN, and 3D CNN [104,105].

The obvious difference in image attenuation between the lung and non-lung regions in a normal lung makes it simple to distinguish between them on a CT scan. To separate the lungs from the non-lung region, early works in lung segmentation used straightforward methods such as numerical approaches, gray-level thresholding, and shape-based approaches.

For natural image processing and medical image analysis, various CNN-based methods have been proposed. Lung nodule segmentation was the focus of early work in this field [103]. Based on the training dataset created using a clustering algorithm-based method, the work in [106] suggested a simple CNN model for lung segmentation. The mean and minimum intensities of the image patch are used as input to the k-means clustering algorithm to divide the CT slices into two groups. A combination of cross-shaped verification, volume intersection, connected component analysis, and patch expansion techniques was used to create the final dataset. A simple one-convolutional layer with six kernels, one maximum pooling layer, and two fully linked layers made up the proposed CNN architecture. Utilizing the generated datasets for training purposes, an eightfold cross-validation approach was used to assess the performance of the CNN models. Through an image decomposition-based filtering method introduced in [107], the researchers have created automatic lung segmentation algorithms that denoise the lung CT images without changing the outlines of the lungs. Wavelet transformation and several morphological techniques were then used to segment the lungs. Finally, the retrieved lung outlines were corrected and smoothed as part of the segmentation refining process using a contour correction approach.

For lung CT segmentation, Khanna et al. [108] developed a Residual U-Net with a false-positive reduction approach. The proposed model consists of a more complex network with residual units, making it easier to extract the distinguishing properties needed for lung segmentation. On the other hand, the work in [109] examined two deep learning models, U-Net and E-Net, to analyze the performance differences between them. The results show that the parenchyma of patients with pulmonary fibrosis can be efficiently and quickly segmented by these models.

In [11], the authors suggested a lung segmentation method based on a U-net architecture that has an expanding path for recovering high-level information and a contracting path for extracting low-level information. The experimental results show that the dice coefficient performance is 0.9502. Another automatic lung segmentation technique in [110] was created using a mask R-CNN methodology that was integrated with both supervised and unsupervised machine learning techniques. This approach provided a quicker and more accurate response compared to the benchmarked methods, with an average runtime of 11.2 s and a segmentation accuracy of 97.68%.

In [111], Setio et al. suggested a multi-view convolutional network employing the training data’s discriminative properties to identify lung nodules. The three-nodule candidate detectors were made to target solid, subsolid, and big nodules. The suggested solution is based on the integration of numerous streams of 2-D ConvNets with a trustworthy classification mechanism. The results tested on the LIDC-IDRI dataset indicate that on average there are four false positive cases per scan, while detection sensitivity is 85.4%. Rather than using the 2D approach, the work in [112] used volumes of interest taken from the LIDC dataset to train a 3D CNN for the autonomous detection of lung nodules. Additionally, a 3D fully convolutional network (FCN) was created using a 3D CNN in order to quickly create the score map for the entire volume in a single run. The discriminating CNN proved effective for quickly producing candidate regions of interest using the proposed FCN-based architecture.

Another automated lung nodule segmentation system was proposed in [15] based on a synergistic mix of deep learning and shape-driven level sets. With the help of the coarse segmentation maps obtained by the deep fully convolutional networks, their method, as depicted in Figure 3, offers coarse-to-fine solution maps. Initially, the level sets’ shape-driven evolution was used to create fine segmentation. Furthermore, the level sets were used to automatically initialize the model using the seed points derived from the deep network’s coarse segmentation.

## 7. Classification Process

**Figure 5:**
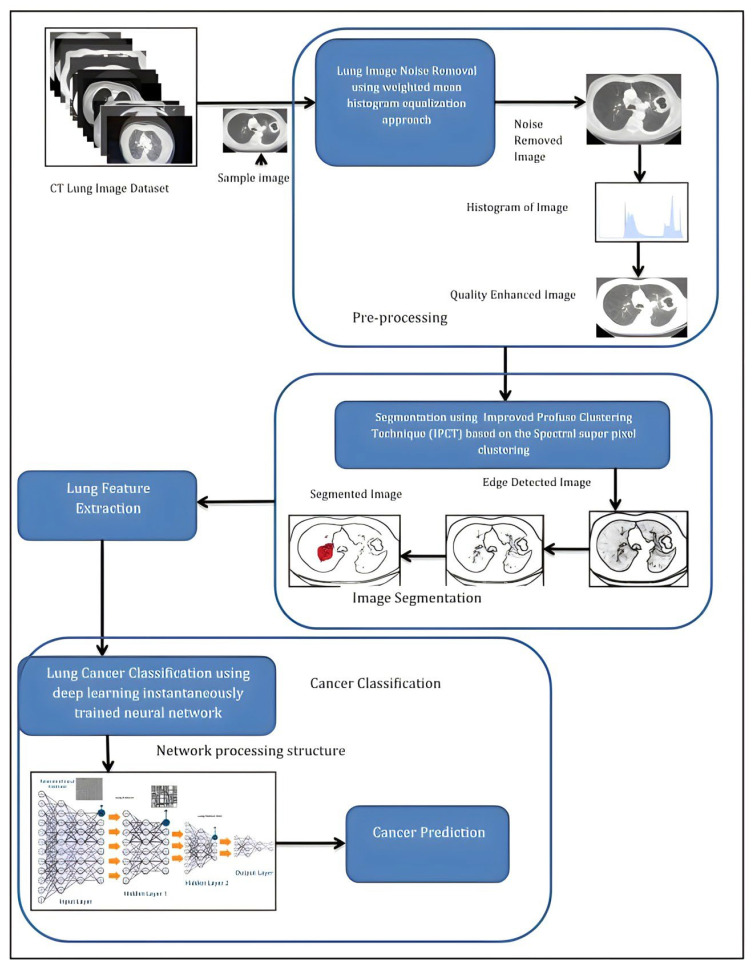
Lung cancer classification structure [57].

In general, deep learning techniques have shown greater promise for disease classification compared to conventional methods. In [57], the authors attempted to improve the quality of lung images and lung cancer diagnosis by reducing the rate of misclassification cases. The CT images were collected from the Cancer Imaging Archive (CIA) dataset, whereby the noises were removed using the weighted mean histogram equalization method. This pre-processing step managed to successfully reduce noise in the images. Furthermore, a new segmentation algorithm was proposed based on an improved profuse clustering technique (IPCT), where the spectral features from the afflicted region were extracted from the segmented images. These characteristics were then used to feed the input into a deep-learning algorithm to detect lung cancer. Figure 5 summarizes the lung cancer classification method used in the study.

Computer-aided diagnostic (CAD) systems have been assisting radiologists in making appropriate conclusions. The accuracy of CAD systems in various medical imaging disciplines is growing dramatically as intelligent deep-learning tools and methodologies have also improved significantly. Among the existing imaging modalities for lung cancer diagnosis, such as Chest X-ray, Computed Tomography (CT) Scan, Positron Emission Tomography (PET) Scan, MRI, and CE-CT (Contrast-Enhanced Computed Tomography), CT images are known to be suitable to capture sufficient information for the stage classification of non-small cell lung cancer. According to the review study on lung nodule classification, a combination of CT images with deep learning algorithms has been successfully applied in many papers to detect malignant tumors [61].

The majority of previous research on lung cancer detection only focused on classifying the nodules as benign or malignant. In [113], Liu et al. suggested a Multi-view Convolutional Neural Network (MV-CNN) for binary and ternary classifications of lung cancer. For both binary and ternary classifications, the performance of the multi-view strategy outperformed the single-view approach. Da Silva and da Silva [114] then proposed another lung nodule classifier that used a combination of deep learning and genetic algorithms to determine whether a nodule is malignant or benign. The sensitivity, specificity, accuracy, and area under the ROC of the suggested technique, tested on the LIDC-IDRI database, were 94.66%, 95.14%, 94.78%, and 0.949, respectively. 

For a 3D counterpart of the previous work, Dey et al. [115] developed a binary classifier for translating 3D pictures to class labels using a four-pathway CNN. A basic 3D CNN, a novel multi-output network, a 3D DenseNet, and an upgraded 3D DenseNet with multi-outputs were incorporated into the proposed CNN. The performance of these four networks was validated using the public LIDC-IDRI dataset and found to outperform the majority of existing approaches.

In [116], the authors described a CNN-based lung cancer classifier that uses two neural networks for the extraction and classification of the cancer features, utilizing FDGPET and CT images. Convolution operators were used to producing low-level features, whereas ReLU and max-pooling down-sampled feature maps were tasked with extracting higher-level features. This work aims to differentiate between the T1–T2 and T3–T4 classes that yield accuracy, recall, specificity, and AUC values of 90%, 47%, 67%, and 0.68, respectively.

The following issues have been identified in the literature based on the developed lung cancer classification algorithms: (a) Binary lung classification algorithms usually do not meet the standard diagnosis of the radiologist and oncologist; (b) An ROI patch-based method takes diagnosis time and requires manual expert involvement; and (c) Standard image processing algorithms failed to segment the lung nodules accurately. Table 3 shows the comparison results.

## 8. Limitations

There are various problems connected with pre-processing in the field of lung cancer identification and diagnosis utilizing deep learning techniques on CT scans, especially when working with heterogeneous datasets. These constraints can have an impact on the classification and segmentation tasks’ accuracy and reliability. Some of the major restrictions are as follows: Due to variances in imaging techniques and equipment, CT scans of lung cancer might vary greatly regarding resolution, slice thickness, contrast, and noise levels. This heterogeneity presents a difficulty for pre-processing procedures, which must successfully handle these disparities to produce consistent outcomes. Failure to address data variability can lead to inferior deep-learning model performance and generalizability. CT pictures can contain artifacts such as motion artifacts, beam hardening, and metal artifacts, which can compromise image quality. These artifacts can generate inconsistencies and distortions in the data, making accurate feature and information extraction challenging for pre-processing procedures. To minimize the impact of artifacts on future classification and segmentation procedures, robust artifact identification and repair algorithms are required. For training, deep learning models for lung cancer detection and diagnosis often require a considerable amount of labeled data. Obtaining appropriate annotations for CT scans, on the other hand, can be difficult and time-consuming, especially for intricate segmentation tasks. The scarcity of labeled data might constrain deep learning model training and evaluation, reducing performance and generalizability. Furthermore, using varied annotation techniques and criteria across datasets can generate differences and biases in the training process. Deep learning models, particularly those based on convolutional neural networks (CNNs), can be computationally expensive, necessitating large amounts of computing power for training and inference. Image resizing, normalization, and augmentation, for example, must strike a balance between maintaining useful information and decreasing computational demands. Inadequate pre-processing procedures may result in either information loss or high computing requirements, affecting the deep learning pipeline’s efficiency and scalability. Most deep-learning studies in lung cancer detection and diagnosis have concentrated on certain populations or datasets that may not effectively represent the diversity of patients and imaging practices. To ensure that deep learning models generalize successfully to varied populations and imaging circumstances, pre-processing techniques must address potential biases and constraints associated with individual datasets. To overcome these pre-processing restrictions, careful evaluation of dataset features, robust algorithmic approaches, and cooperation across various universities are required.

## 9. Discussion

Imaging technologies such as computed tomography (CT), magnetic resonance imaging (MRI), and positron emission tomography (PET) scans are used to diagnose lung cancer. Once a doctor concludes that there is reason to suspect cancer, he or she may perform additional tests such as a biopsy, ultrasound, mediastinoscopy, thoracoscopy, or wedge resection. Lung cancer is a complex disease with numerous origins, tumor kinds, and symptoms, making a precise diagnosis crucial for the best possible prognosis. A medical Center with experience diagnosing and treating lung cancer is more likely to correctly identify the source of symptoms. When cancer is detected, it is staged, which informs the patient and doctors about the size of the tumor and where it has spread beyond the initial site. The use of imaging technologies, such as CT scans, which employ X-rays to make cross-sectional images of the chest, is the initial step in diagnosing lung cancer. MRI scans provide detailed images of soft tissue using radio waves and strong magnets. Similar to CT scans, MRI scans can generate detailed images of the tissue in the chest cavity. They are mostly used to determine whether lung cancer has progressed beyond its initial site. PET scans employ injected fluorodeoxyglucose (FDG) to reveal cancer cells. They can also be used to determine whether cancer has spread beyond the initial spot. PET/CT scans integrate both technologies to provide the clinician with a more detailed image.

In Part 4, it is discussed how lung cancer detection methods are evolving. The techniques include spiral CT scans, deep learning, convolutional neural networks, sputum cytology, white light bronchoscopy, chest radiographs, computer-aided diagnosis, and CT imaging. One of the top imaging methods for detecting lung cancer is CT imaging. It can find lung cancer nodules that are both suspected and undiscovered. However, it might be challenging to effectively identify malignant cells due to the variation in intensity in CT scan pictures and anatomical structure misjudgments by physicians and radiologists. CAD systems are created to aid radiologists and medical professionals in precisely identifying cancer. These systems analyze CT scans to assist in the diagnosis of lung cancer using image processing methods and machine learning algorithms. To obtain higher detection accuracy, additional enhancements are required, as some CAD systems may not be accurate enough. X-rays of the chest are less expensive and more widely available than CT scans. Compared to CT scans, they are less sensitive but have lower radiation doses. The high false-positive rate of chest CT, which necessitates extra follow-up and invasive tests, is a drawback. Sputum samples are examined for the presence of malignant cells by sputum cytology. The ability to detect core tumors coming from bigger bronchi is particularly advantageous. Sputum cytology is inadequately sensitive or accurate for routine use in the diagnosis of lung cancer and has limits in the detection of tiny adenocarcinomas. To make a conclusive histological diagnosis of lung cancer, WLB is a frequently employed diagnostic method. Nevertheless, it has limitations in identifying pre-malignant lesions, and a precise diagnosis may call for additional tests or biopsies. Although they have poorer sensitivity for tumors that are centrally situated, spiral CT scans have demonstrated improved diagnostic capability for peripherally tiny tumors. Spiral CT scans have a significant false-positive rate, which can result in invasive and expensive procedures being performed on people who do not have lung cancer. CNNs have demonstrated promise in radiology and lung cancer diagnosis. They can enhance radiologists’ performance and help spot nodules and masses on chest radiographs. However, special consideration should be given to achieving proper identification and discrimination between benign and malignant nodules because the sensitivity and false-positive rate of these models can vary. Each of the aforementioned techniques or methods has its own drawbacks, including the potential for false positives, lesser sensitivity for specific tumor types, challenges with precisely identifying malignant cells, and the requirement for additional advancements to increase accuracy. To get beyond these obstacles and enhance the early identification and treatment of lung cancer, advancements and research in these fields must continue.

## 10. Conclusions

The review paper examines the body of knowledge on lung cancer diagnosis and presents several recommendations for further study in the area. The authors stress the requirement for additional lung image data from multiple imaging modalities, such as MRI and ultrasound, in addition to the significance of disclosing private datasets to enable comparison and research collaboration. The segmentation of big solid nodules is one area that has been specifically mentioned. This difficult task needs more investigation. The scientists also suggest creating a lung cancer detection model that can distinguish between early benign nodules and small malignant lesions, which would dramatically improve early identification and treatment. To increase the effectiveness of automated tumor identification, it is also recommended that deep features acquired from lung scan images be combined with additional patient data, including medical history and genetic reports. This all-encompassing method might offer a more precise diagnosis of the illness. The authors suggest using various pre-processing techniques and filters to improve image quality, such as edge-preserving methods and harmony search to improve grayscale image quality. These techniques can aid in improved picture analysis and more trustworthy diagnostic outcomes. A successful proposal for remote lung cancer detection serves as motivation for the authors’ further suggestion to investigate the use of cloud computing technology for machine learning-based remote diagnosis of lung cancer. Large volumes of medical data can be processed and analyzed effectively by using cloud computing. The scientists suggest using a cat swarm-optimized deep belief network to extract features from lung medical images since it may perform better in feature extraction and classification tasks.

## 11. Future Research Direction

Future research should focus on creating standardized pre-processing methods that can deal with CT picture variability and enhance the precision and dependability of lung cancer detection and diagnosis using deep learning methods. The review report emphasizes the need for improvements in lung cancer diagnosis in its conclusion. Future research directions are suggested in many different areas, including data accessibility, segmentation, early detection, integration of patient data, picture quality enhancement, cloud computing, feature extraction, and standardization. Researchers can increase the precision, effectiveness, and dependability of lung cancer diagnosis by focusing on these issues, which will ultimately help patients all around the world.

## Figures and Tables

**Table 1:** Comparison of imaging methods.

Imaging Method	Applications	Pros	Cons
X-ray	Rib fracture detection, pneumonia detection, and lung cancer screening	Quick, affordable, and broadly accessible	Limited sensitivity and specificity, which could miss lung cancer in its early stages.
CT (computed tomography)	Lung cancer screening, lung disease diagnosis, lung cancer extent assessment, and pulmonary embolism detection	Detects tiny or early-stage lung malignancies with high resolution and sensitivity and is helpful for examining lung nodules.	High radiation dose, potential need for contrast agent, and high cost
Ultrasound	Identifying pleural effusions, directing thoracentesis, and assessing diaphragm performance	Non-invasive, radiation-free, and usable at the bedside	Operator-dependent, limited capacity to scan lung parenchyma; gas or bone obstructions possible.
MRI (magnetic resonance imaging)	Evaluation of the invasion of lung cancer, diagnosis of pulmonary embolism, and evaluation of lung function	Good soft tissue contrast, little radiation exposure, and the ability to assess lung function	Long scan times, little availability, high cost, and potential need for contrast agents
PET-CT (positron emission tomography—computed tomography)	Assessing lung cancer, staging lung cancer, observing treatment results, and spotting recurrences of cancer	High sensitivity for detecting cancer, early cancer detection capability, and anatomical and functional information provided	False positives caused by inflammation or infection, high radiation dose, price, and possible requirement for fasting before the scan

**Table 2:** Summarization of references (latest 5 years).

Reference	Year	Application	Method Used and Dataset	Pros	Cons
[21]	2018	CT Lung image	Level-set approach for joint image segmentation and registration and CT image dataset	The technique allows for the simultaneous processing of these tasks by combining image segmentation and registration into an identical framework	The technique is only directly applicable to CT lung imaging, excluding additional imaging modalities or anatomical locations
[43]	2018	Segmenting a CT image of the lung	Deep neural networks and the Lung Image Database	Increases efficiency and accuracy	A small sample size
[32]	2018	Identification of lung nodules	Dense Convolutional Binary-Tree Networks and LIDC-IDRI	Achieved great accuracy in classifying lung nodules.	Restricted by the lack of readily accessible, large training datasets
[33]	2018	DL-CAD, or deep learning-based computer-aided diagnosis, is a method for identifying and classifying lung nodules	Deep Learning-based Computer-Aided Diagnosis System	Achieved great accuracy in lung nodule detection and characterization and showed promise for enhancing radiologists’ performance	It is constrained by the availability of huge training datasets, and it might not work well on images with poor contrast or strange morphology
[34]	2018	Reduced likelihood of false-positive lung nodule identification	Deep 3D Residual CNN and CT data	Reduced false positives in the detection of lung nodules with excellent accuracy	Depending on the quality of the input photographs, the algorithm may not function properly on images with poor contrast or strange morphology
[26]	2019	Identification and classification of lung cancer	Deep Convolutional Neural Networks (CNNs), en-source data sets, and multicenter data sets have been used.	Displayed expert-level proficiency in the spotting and sizing of lung cancer	Requires massive datasets for training and may struggle with images that have poor contrast or a strange shape
[37]	2019	Lung adenocarcinoma	Deep learning on CT images and imageNet	Predicting the status of the EGFR mutation	A small sample size
[44]	2019	CT imaging for the identification of lung cancer	Improved profuse clustering and deep learning using instantaneously trained neural networks and CT image datasets	Increased precision and fewer false positives	Large datasets and specialized knowledge are required
[27]	2019	Artificial neural network-based lung cancer detection	Artificial Neural Network (ANN) and our ANN established, trained, and validated using a data set, whose title is “survey lung cancer”	Classification of benign and malignant nodules with good accuracy	Limited by the poor interpretability of the model and the caliber of the input images
[57]	2019	CT imaging for the identification of lung cancer	Improved profuse clustering and deep learning instantaneously trained neural networks and the lung CT images are collected from the cancer imaging archive (CIA) dataset	Increased precision and fewer false positives	Large datasets and specialized knowledge are required
[58]	2019	3D CT images used to diagnose a lung nodule	Deep convolutional neural networks and LIDC-IDRI	Improved diagnostic precision and decreased inter-observer variability	Large datasets and specialized knowledge are required
[40]	2019	Screening for lung cancer	Three-dimensional deep learning on low-dose CT and NLST datasets	High efficiency and accuracy	Huge volumes of training data are necessary
[29]	2019	Finding the stage of lung cancer	Double Convolutional Neural Network (CNN) And CT images from the initial dataset so that the training of the CDNN could be focused	Achieved great accuracy in identifying the stage of lung cancer	Depending on the quality of the input photographs, the algorithm may not function properly on images with poor contrast or strange morphology
[30]	2019	Classification and identification of pulmonary nodules	CNN-based nodule-size-adaptive detection and classification and the Tianchi AI dataset	The detection and classification of pulmonary nodules were carried out with great accuracy	Depending on the quality of the input photographs, the algorithm may not function properly on images with poor contrast or strange morphology
[39]	2019	Identification, segmentation, and classification of pulmonary nodules	Deep learning on CT images and NLST	Increases efficiency and accuracy	A small sample size
[31]	2019	Image categorization for the lungs	Inception-v3 Transfer Learning Model and ImageNet dataset	Good classification accuracy for pulmonary images.	Restricted by the lack of readily accessible, large training datasets.
[37]	2019	Lung adenocarcinoma	Deep learning on CT images and ImageNet datasets	Predicting the status of the EGFR mutation	A small sample size
[49]	2020	Finding pulmonary nodules	Computer-aided diagnosis (CAD) system with deep learning and CT image dataset	Extremely sensitive and specific	Large volumes of training data are required
[51]	2020	Lung cancer prediction	Deep learning framework and eCT image dataset	Early, non-intrusive detection	Limited information and possible false positives
[55]	2020	Lung cancer	Radiomics and deep learning	Improved prognosis and diagnosis accuracy	Large datasets and specialized knowledge are required
[38]	2021	Lung adenocarcinoma	Deep learning on CT images	Predicting survival and subtype classification	A small sample size
[61]	2021	Classification of lung nodules	Deep learning on CT images	High accuracy and efficiency	A small sample size
[42]	2021	Non-small-cell lung cancer	Deep learning on CT images and immunotherapy datasets	PD-L1 expression and EGFR mutation prediction	A small sample size
[47]	2021	Evaluation and forecasting of PD-L1 expression	Deep learning on CT images and the PD-L1 expression dataset	Enhanced precision and non-invasive	Large volumes of training data are required
[48]	2021	Extraction of information on lung cancer staging	Deep learning approach and CT dataset	Automated and saving time	Limited information and possible mistakes
[56]	2021	Prediction of cardiovascular disease risk from CT of lung cancer	Deep learning and NLST and MGH datasets	Enhanced early detection and risk assessment	Restricted by computing power and labeled data accessibility
[59]	2021	Using an electronic nasal device and identify lung cancer	Weighted discriminative extreme learning machine design and lung cancer datasets and public datasets	Invasive-free detection technique	Only able to identify lung cancer in its first stages
[60]	2021	PET/CT imaging for non-small cell lung cancer detection	Multimodality attention-guided 3-D detection using deep learning and a private dataset	Improved diagnostic precision and fewer false positives	Large datasets and specialized knowledge are required
[50]	2022	Prediction of benign, preinvasive, and invasive lung nodules	Machine learning and CT dataset	Enhanced accuracy and early detection potential	Large training data requirements and the risk for false positives
[41]	2022	Screening for lung cancer	Deep learning on mobile low-dose CT and CT image datasets	Improved access in areas with limited resources	A small sample size
[43]	2022	Identification and detection of pulmonary nodules	Deep learning on CT image and CT lung datasets	Increases efficiency and accuracy	A small sample size
[28]	2022	CT imaging for the identification of lung cancer	Improved Profuse Clustering and Deep Learning Instantaneously Trained Neural Networks and images of CT scans	More accuracy compared to conventional clustering-based approaches and shorter training times	Compared to other deep learning models, it requires more parameters and more training time
[52]	2023	Prediction with CT scan and histopathological images	Six different deep learning algorithms like Convolutional Neural Network (CNN), CNN Gradient Descent (CNN GD), VGG-16, VGG-19, Inception V3, and Resnet-50.	Using the CNN GD provides the ability to learn from training data over time and its efficient cost function within gradient descent, which continuously assesses accuracy during parameter updates	The lack of integration with fuzzy genetic optimization techniques, which could potentially enhance the methodology’s performance and effectiveness
[53]	2023	A novel method called Cancer Cell Detection uses Hybrid Neural Network (CCDCHNN) to extract features from the CT scan images using deep neural networks	The approach in this research suggests a sophisticated 3DCNN with an RNN algorithm for classifying cancerous lung nodules. The system makes use of the LUNA 16 database.	The proposed improved model provides single 3D-CNN and RNN classifications with high selectivity, sensitivity, and accuracy	Enhanced efficiency by integrating big-data analytics and cascaded classifiers, which are not currently utilized in the proposed approach
[54]	2023	The proposed model used several convolutional layers to perform the detection task from CT scan imaging	The study developed a CNN-based model with 99.45% accuracy for early lung cancer prediction using CT scan images. The dataset used was the IQ-OTH/NCCD-lung cancer dataset from Kaggle	The proposed CNN-based model achieved a high accuracy rate of 99.45% for early lung cancer prediction and successfully reduced false positives	The major gap in this work is the limited number of epochs used during training

**Table 3:** Performance comparisons.

S. No	Method	Accuracy (%)
1	Lung CT image segmentation using deep neural networks [11].	95%
2	Biological and gene expression data using a fuzzy preference-based rough set [13].	96.19%
3	Lung cancer detection using an artificial neural network [27].	96.67%
4	SegChaNet: A novel model for lung cancer segmentation in CT scans [28].	98.48%
5	Optimal deep learning model for classification of lung cancer on CT images [44].	94.56%
6	Lung cancer prediction using a deep learning framework [51].	99.52%
7	An effective approach for CT lung segmentation using mask region-based convolutional neural networks [110].	97.68%

## Data Availability

Not applicable.
